# A young nonsmoker male patient with breathlessness

**DOI:** 10.4103/0970-2113.56346

**Published:** 2009

**Authors:** Ramakant Dixit, Sidharth Sharma, Manoj Arya

**Affiliations:** *Department of Pulmonary Medicine, J. L. N. Medical College, Ajmer, India*

## CASE SUMMARY

A 32-year-old man presented with a medical history of weight loss, recurrent diarrhea and breathlessness for last 1 year. A recent episode of mild hemoptysis prompted him to consult us. He had no history of fever, cough with expectoration, pneumonias or other medical illness in recent or remote past. His family history did not reveal any hereditary lung diseases. He had never smoked or used intravenous drugs but disclosed frequent unprotected extramarital sex. He worked as shopkeeper, had never been exposed to fumes, dust, asbestos, silica, etc. There was no history of urticaria, eye disease, joint symptoms, etc.

On general physical examination, he had body mass index 15.4, pallor, seborrhoic dermatitis over major parts of body, oral ulcers with thrush, tachypnea (24 breaths/min) and hypoxemia (pulse oxymetric saturation 90% while breathing room air). Physical examination was unremarkable for icterus, cyanosis, clubbing, edema, joint abnormality, etc.

Examination of respiratory system revealed emphysematous chest with distant breath sounds bilaterally and hyperresonence at right lower lung fields. The findings of cardiac, gastrointestinal and neurological examinations were essentially normal.

**Figure 1:**
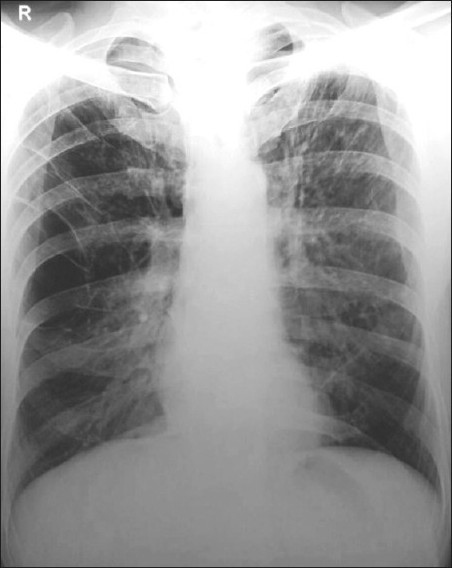
Skiagram chest showing hyperinflated lung fields with a large bullous lesion on the right side

His investigations revealed hemoglobin 10 g%, with normal blood counts and organ functions. The serological examination for human immunodeficiency virus (HIV) was positive on two different enzyme-linked immunosorbent assay (ELISA) kits. The CD4^+^ T lymphocytes count was 120 cells/mm^3^. The induced sputum was negative for acid-fast bacilli, bacteria or fungi. The X-ray chest posteroanterior view revealed bilateral hyperinflated lung fields with a large bullous lesion on the right side [Figure 1].

**Figure 2:**
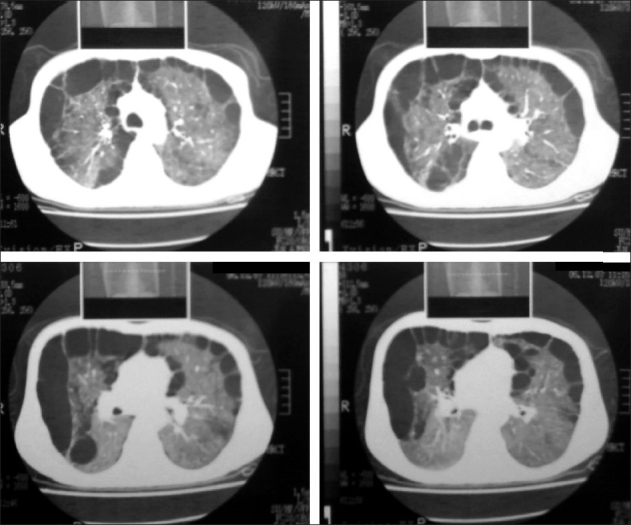
HRCT chest showing emphysematous lungs with multiple, medium- to large-sized bullous lesions

Pulmonary function tests revealed forced vital capacity of 1.69 L (63% predicted), forced expiratory volume (FEV) 1 of 1.03 L (45% of predicted), FEV1/FVC ratio of 70% and peak expiratory flow 2.56 L/s. Facility to determine diffusion of carbon monoxide and plethysmography was not available. Fibreoptic bronchoscopy to search cause for haemoptysis did not reveal any endobronchial abnormality and laboratory examination of bronchial washings and lavage was normal with no evidence of *Pneumocystis carinii* pneumonia. The result of a tuberculin skin test was also negative. High-resolution computerized tomography of the chest revealed bilateral emphysematous lungs with multiple, medium to large, peripheral and paraseptal bullous formation with loss of normal lung parenchyma in upper and middle lobe on the right side and upper lobe and lingual on the left side, sparing bilateral lower lobes. There was no cardiomegaly, significant lymphadenopathy, mediastinal, vascular or bone cage abnormality, etc. [Figure 2].

A comprehensive metabolic panel including a1-antitrypsin (A1AT) level (175 mg/dl) and angiotensin-converting enzyme level (28 U/L) was also normal.

Patient was treated with bronchodilators (inhaled beta-2 agonist and oral theophyllin), antiretroviral therapy (including lamivudine, stavudine and nevirapine) and short-term antifungal therapy with fluconazole, and was then put on prophylactic cotrimoxazole. At present, he is clinically stable, had no opportunistic infection and doing his job well for last more than three years.

## WHAT IS YOUR DIAGNOSIS?

ANSWER

Bullous emphysema/bullous lung disease secondary to human immunodeficiency virus (HIV) infection.

## DISCUSSION

Our patient presented with extensive bullous emphysema with no history of smoking. Although cigarette smoking is the major risk factor for the development of chronic obstructive pulmonary disease (COPD), not all smokers develops COPD, and such patients deserve a workup for one of the less common causes of emphysema. These conditions includes A1AT deficiency, connective tissue disease (Cutis laxa, Marfan syndrome, Eehler-Danlos syndrome), intravenous drug abuse (methylphenidate, cocaine or talc), HIV infection, hypocomplementemic urticarial vasculitis syndrome, malnutrition and several rare metabolic disorders (Salla disease, Menke syndrome).[1] Other differential diagnoses of bullous emphysema and bullous lung disease include uncommon causes such as autoimmune diseases (Sjogren disease, wegners granulomatosis disease and multisystem autoimmune dysfunction), bullous sarcoidosis, Birt-Hog-Dube syndrome, neurofibromatosis, placental transmogrification of the lung, fabry disease, idiopathic giant bullous emphysema, etc.[2] Almost all these conditions have systemic manifestation with characteristic features including early onset, liver dysfunction, vasculitis, skin and joint manifestations, lymphadenopathy, etc. The evaluation of bullous emphysema in nonsmokers therefore begins with a detailed medical history, including age of onset of the disease and a physical examination, including determination of the presence of extrapulmonary symptoms or signs and measurement of A1AT level.[3] On the basis of clinical and laboratory data, our patient had none other than HIV infection as a cause for bullous emphysema.

HIV infection has recently emerged as an important cause of bullous emphysema and bullous lung disease. HIV infection is associated with COPD and airways abnormalities, including features of emphysema, chronic bronchitis, nonspecific airways abnormalities and bronchial hyper-responsiveness.[4] In industrialized countries, a 15% to 42% prevalence of HIV-related pulmonary emphysema has been observed, which is much higher than the estimated 1.8% prevalence in the general population.[5] In the era of highly active antiretroviral therapy (HAART) also, HIV infection has been described as an independent risk factor for COPD after adjusting for age, race/ethnicity, pack years of smoking, injection drug use and alcohol abuse.[6]

The risk factors for COPD among HIV-positive patient are several and the most potent among them are cigarette smoking. An accelerated progression of emphysema has been reported in young HIV-positive individuals who are smokers.[5] Additional potential risk factor includes inhaled and intravenous substance abuse, occupational or environmental exposures, low socioeconomic status, repeated pulmonary infection (especially *Pneumocystis jiroveci* colonization of respiratory tract, chronic viral infection such as with adenovirus, etc) and HIV infection itself.[4,7] In our case, these risk factors were not present, suggesting HIV infection itself causing emphysematous changes. Similar to our case, Diaz *et al.*[8] reported four patients without history of pneumonia, other pulmonary opportunistic infections or other known causes for emphysema, showing marked abnormal pulmonary function with air-trapping, hyperinflation, decreased diffusion capacity and emphysema-like bullous changes on high-resolution computerized tomography, suggesting a distinct role of HIV infection. In one autopsy study of patients with AIDS, infection of macrophages by the HIV virus resulted in the upregulation of matrix metalloproteases in neighboring infected macrophages and was strongly associated with loss of alveolar wall.[9] CD8^+^ T cells are also believed to play a critical role in the development of COPD, as HIV infection can result in the intense infiltration of CD8^+^ T cells into the lung, which have been shown to secrete large amounts of interferon-γ.[4] While it is unclear whether this lymphocytic alveolitis and expression of interferon-g play any role in the COPD development in HIV-positive individuals, the overexpression of interferon-g has been shown to cause emphysema in animal models.[10] Further, HIV-positive patients have evidence of abnormal systemic and lung oxidant/antioxidant balance and this oxidative stress may play a key role in the development and progression of COPD.[4] Recent developments in the pathobiology of HIV-associated emphysema has helped in our understanding of how HIV infection may affect cytotoxic lymphocyte activation, lung capillary endothelial cell injury and apoptosis, sphingolipid imbalance and oxidative stress in the lung. A better understanding of the pathogenesis of HIV-associated pulmonary emphysema may provide clues and therapeutic targets that have broader application in this disease, including cigarette smoke-induced emphysema.[11]

## CONCLUSION

HIV-positive patients appear to have an increased risk for COPD. Although, some of the increased COPD may be attributed to the smoking and drug abuse in HIV-positive patients, the apparent risk for COPD remains high in such patients even after controlling for these and other potential confounders.[5,6] COPD is likely to contribute substantially to the morbidity and mortality of HIV-positive patients in the era of HAART. Because COPD is irreversible and progressive in nature, it could compromise the benefits of HAART in resource-poor settings. Finally, health care providers should be aware of the likelihood of increased COPD among HIV-positive patients.

## References

[1] Lee P, Gildea TR, Stoller JK (2002). Emphysema in nonsmokers: Alpha 1-antitrypsin deficiency and other causes. Cleveland Clin J Med.

[2] Teramoto S, Fukuchi Y (1996). Bullous emphysema. Curr Opin Pulm Med.

[3] Mireles-Cabodevila E, Sahi H, Farver C, Mohammed TL, Culver DA (2007). A young patient with a minimal smoking history presents with bullous emphysema and recurrent pneumothorax. Chest.

[4] Crothers K (2007). Chronic obstructive pulmonary disease in patients who have HIV infection. Clin Chest Med.

[5] Diaz PT, King MA, Pacht ER, Wewers MD, Gadek JE, Nagaraja HN (2000). Increased susceptibility to pulmonary emphysema among HIV-seropositive smokers. Ann Intern Med.

[6] Crothers K, Butt AA, Gibert CL, Rodriguez-Barradas MC, Crystal S, Justice AC (2006). Increased COPD among HIV-positive compared to HIV-negative veterans. Chest.

[7] Morris A, Sciurba FC, Lebedeva IP, Githaiga A, Elliott WM, Hogg JC (2004). Association of chronic obstructive pulmonary disease severity and Pneumocystis colonization. Am J Respir Crit Care Med.

[8] Diaz PT, Clanton TL, Pacht ER (1992). Emphysema-like pulmonary disease associated with human immunodeficiency virus infection. Ann Intern Med.

[9] Yearsley MM, Diaz PT, Knoell D, Nuovo GJ (2005). Correlation of HIV-1 detection and histology in AIDS-associated emphysema. Diagn Mol Pathol.

[10] Wang Z, Zheng T, Zhu Z, Homer RJ, Riese RJ, Chapman HA (2000). Interferon gamma induction of pulmonary emphysema in the adult murine lung. J Exp Med.

[11] Petrache I, Diab K, Knox KS, Teigg HL, Stephen RS, Flores S (2008). HIV associated pulmonary emphysema: A review of the literature and inquiry into its mechanism. Thorax.

